# The Impact of Tank Disinfectants on the Development of Microbiota in Gilthead Seabream (*Sparus aurata*) Larviculture Systems

**DOI:** 10.3390/microorganisms13061359

**Published:** 2025-06-11

**Authors:** Georgia Apostolopoulou, Naima Bel Mokhtar, Elias Asimakis, Eva Dionyssopoulou, Kosmas Toskas, George Koumoundouros, George Tsiamis, Panagiota Stathopoulou

**Affiliations:** 1 Laboratory of Systems Microbiology and Applied Genomics, Department of Sustainable Agriculture, University of Patras, 30100 Agrinio, Greece; gogoapostolopoyloy1@gmail.com (G.A.); naima1503@gmail.com (N.B.M.); eliasasim@gmail.com (E.A.); ediony@gmail.com (E.D.); gtsiamis@upatras.gr (G.T.); 2 Avramar Aquaculture SA, 19.3 km Markopoulou Paianias Avenue, 19002 Paiania, Greece; k.toskas@avramar.eu; 3 Biology Department, University of Crete, 71003 Heraklion, Greece; gkoumound@uoc.gr

**Keywords:** 16S rRNA, bacterial diversity, food security, tank disinfection, larviculture, opportunistic infection, resident bacteria

## Abstract

Aquaculture play a vital role in enhancing human nutrition by producing commercially valuable fish, with gilthead seabream (*Sparus aurata*) being a key species in the Mediterranean region. In seabream larviculture, disinfection is commonly used to control pathogens and prevent microbial imbalances. However, this process may also remove beneficial microbiota that contribute to ecosystem stability. This study aims to investigate the impact of tank disinfection operations on the bacterial communities associated with seabream larvae and their rearing water in a commercial hatchery using 16S rRNA amplicon sequencing. For further comparison, the bacterial communities present in eggs and feed were also analyzed for comparison. Results showed that the use of different disinfectants significantly altered the bacterial composition of the larvae, while the duration of the dry period had no measurable effect. Across all larval samples, the phylum Pseudomonadota dominated, with members of the genus *Psychrobacter* consistently detected regardless of disinfection treatment. This suggests that *Psychrobacter* may be transmitted from eggs or acquired through the feed, mainly rotifers and *Artemia nauplii*. In contrast, the bacterial communities in the rearing water were more diverse and showed only minor differences in relative abundance across disinfection methods.

## 1. Introduction

Gilthead seabream (*Sparus aurata*) is the leading species in Mediterranean aquaculture due to its high market value and adaptability to farming conditions. Greece, a major global producer, accounts for 20% of international production and contributed 58% of the 210,247 tonnes of seabream and seabass produced in 2023 [1]. Seabream alone makes up 54% of Greece’s national seafood output [1]. Over the past 20 years, seabream production has grown in quantity and improved in quality, making a significant contribution to addressing the challenges related to nutrition and food security [2,3,4]. Larviculture, the process of growing fish larvae to a size appropriate for transfer to bigger rearing systems, is a crucial and challenging stage of fish farming [5,6,7]. Despite the advances in adult fish production, larviculture remains a major bottleneck, as it establishes the foundation for the entire production cycle. Infectious disease outbreaks during this phase present a significant economic risk and can threaten the stability of the whole system [8,9]. During this phase, the larvae interact with feed and discarded waste, creating conditions that promote the growth of beneficial, neutral, and potentially harmful microbes [10,11,12]. Beneficial symbiotic bacteria are critical to fish health, development, and survival, as they contribute to vital processes, including digestion and protection against pathogens [13,14,15]. Like humans and other animals, fish also host potentially pathogenic and non-pathogenic commensal bacteria. An imbalance in their microbiota can result in the emergence of pathogenic species and increase the risk of disease [16,17].

Maintaining a healthy microbiome is essential for the overall quality and productivity of fish larviculture [18]. Disinfection is usually the initial step in the treatment of the rearing tanks prior to egg introduction. This step aims to eliminate bacteria, viruses, and other microorganisms that are likely to cause diseases and affect the health of the reared fish. Therefore, it is crucial to disinfect the rearing tanks regularly, following the safety and hygiene standards applicable in the aquaculture industry [19].

A variety of disinfectants are frequently employed in aquaculture, which can be categorized based on their mode of application into synthetic chemical compounds and physical methods. Chlorination, the primary process used in chemical disinfection, is a widely used technique that may eradicate a variety of bacterial species, viruses, and parasites [20]. Chlorine works on bacteria by a reversible process of cell membrane penetration [21,22] and is especially effective in inactivating antibiotic-resistant pathogens [23]. However, it may cause controlled disinfection by-products (DBPs) to develop, which may be detrimental to fish [24,25,26]. After chlorination, it is crucial to neutralize and remove residual chlorine to prevent toxicity to aquatic organisms (especially fish eggs and larvae). Neutralization with sodium thiosulfate is the most common method in aquaculture. After neutralization, a thorough rinse with clean water removes any chemical residue. On the other hand, hydrogen peroxide (H_2_O_2_, HP) is a low-risk compound that breaks down relatively quickly into oxygen and water without leaving harmful disinfection by-products [27,28]. By generating hydroxyl radicals, it is a very effective chemical disinfectant used for fish, water, and feed [29,30,31,32,33]. Another commonly used chemical in aquaculture systems is peracetic acid (PAA). It is thought to be very effective against pathogens, especially bacteria, and environmentally neutral because it hydrolyses quickly to H_2_O_2_ and acetic acid and breaks down into biodegradable residues [34]. It can be utilized to enhance fish tank water quality in terms of reduced ammonium levels without disturbing microbial populations [35]. Peroxymonosulfate (PMS) is an oxidative chemical disinfectant that can inactivate non-enveloped viruses [21]. It can be used under controlled concentrations to disinfect the external surfaces of fish from parasites or eliminate bacterial and fungal pathogens [36]. The typical duration for the mentioned disinfectants is 5–15 min for eggs, 15–30 min for tanks, and up to 1 h for water treatments. Among physical disinfection methods, UV-C radiation displays the least negative effects on the fish microbiome. However, depending on the duration of its application, it can have an impact on phytoplankton, which is used as a background during the larval rearing phase [26,37,38,39].

Hence, the selection of an appropriate disinfectant is contingent upon several factors: (1) the type of aquaculture, since freshwater environments have different disinfection requirements than marine ones; (2) the species of fish, since many of them have varying sensitivity levels to particular disinfectants; (3) targeted disinfection, in which the disinfectant is effective against a particular contaminant or disease; and (4) the environmental effects, which must be considered to determine whether certain disinfectants can be environmentally harmful [40].

Disinfection in larviculture is a widely used but blunt tool, often implemented with limited understanding of its long-term effects on microbial ecology. Previous studies have focused on studying the dynamics of *S. aurata* larval-associated bacterial communities according to water treatment, developmental stages, and potential disease associations [41,42,43,44]. However, there remains a notable gap in understanding the specific impact of tank disinfection practices on both larval and rearing water microbiomes. To address this gap, this study aimed to examine the dynamics of the bacterial communities in both *S. aurata* larvae and rearing water from tanks subjected to different disinfection methods within an industrial production facility. The analysis of the bacterial communities was performed using high-throughput Illumina (San Diego, CA, USA) and ONT MinION (Oxford, UK) sequencing of 16S rRNA gene amplicons. To better understand the impact of the different types of disinfection on the larval and rearing water microbiome, we also examined the bacterial communities present in the provided feed. Additionally, fish eggs were also sampled and compared to the larvae to examine the evolution of the bacterial communities across developmental stages. Understanding the impact of different tank disinfection operations on the microbiome of seabream larvae and their environment could potentially lead to the adaptation of novel disinfection strategies by the aquaculture industry, reduce severe pathogen outbreaks, and eventually enhance production yields.

## 2. Materials and Methods

### 2.1. Experimental Setup and Sample Collection

The experiment was carried out under commercial farming conditions of *S. aurata* between January and June 2020 in a commercial hatchery (Vonitsa, Western Greece). This company is registered (registration number GGN 5200700699992) for aquaculture production in Greece and has secured a GLOBAL G.A.P quality certification. Three different disinfection operations were applied to the larval rearing tanks. After 24 h chlorination period, the tanks underwent one of the three disinfection treatments: (1) nebulization-based disinfection using a commercial product formulated with PAA (1–2.5%), and hydrogen peroxide (H_2_O_2_, HP) (10–20%) (Dis1), (2) disinfection using a commercial product formulated with potassium peroxymonosulfate (PMS) (21.41%) and sodium chloride (NaCl, SC) (1.5%) followed by a one-week dry period (Dis2), or a 50-day dry period (Dis3) (Table 1). After disinfecting tanks (10–30 min) with commercial products, thorough cleaning and rinsing with clean water are essential to ensure no harmful residues remain before rearing aquatic organisms.

Fertilized eggs were derived from broodstock, disinfected, maintained at the same aquaculture facility, and placed in disinfected rearing tanks with a density of around 100 eggs/L. Eggs were surface disinfected, using iodophors, the most used, effective, and relatively gentle approach. Disinfection does not penetrate the egg interior; only surface pathogens are targeted. The hatching and breeding were carried out in a continuous water flow-through using natural sea water (treated with mechanical filtration and UV light sterilization) [45]. The feeding regime was as follows: microalgae (*Chlorella*) were added from 1- to 8-days post-hatching to support the early microbial environment; rotifers were introduced as the primary live feed from 9- to 14-dph; and *Artemia* were introduced at 15-dph. Larvae and their rearing water were collected at various time points: 3-, 8-, 11-, 14-, and 18-dph by the company and were provided post-mortem for analysis. Due to the small size of larvae, the analysis was conducted on the whole body. Samples of larvae in their surrounding water were collected from each tank using a 5 mL sterile plastic pipette to minimize cross-contamination and transferred to sterile containers. To prepare each larval sample, 1.5 mL of water containing larvae was centrifuged at 7000× *g* for 1 min. After the removal of the supernatant, the resulting pelleted material was subjected to DNA extraction. Samples of rearing water (1 L) were also collected at each time point in sterile flasks. These samples were filtered through 0.22 μm pore-sized membrane filters, which were used for DNA extraction. Three replicates were prepared for each larval and water sample. To investigate the contribution of different factors on the initial establishment of the larval bacteriome, samples of eggs collected 1–3 days before hatching (dph) (n = 36) as well as feed samples, including *Chlorella* (n = 14), rotifers (n = 13), and *Artemia nauplii* (n = 7), were also collected and underwent a similar process to the larval samples.

### 2.2. DNA Extraction, and 16S rRNA Gene Amplification

Total DNA extraction from all the samples, larvae, water filters, eggs, and feed were performed following a modified CTAB protocol [46]. The quality of DNA preparations and the concentration of double-stranded DNA were estimated using a Q5000 micro-volume UV Vis spectrophotometer (Quawell Technology, San Jose, CA, USA). DNA samples were preserved in 1.5 mL Eppendorf tubes at −20 °C until further use.

Polymerase chain reaction (PCR) amplification of the V3–V4 region of the 16S rRNA gene (~460 bp) was performed on the larvae, rearing water, and feed DNA samples using barcoded fusion primers U341F–MiSeq 5′-CCTACGGGRSGCAGCAG-3′ and 805R-MiSeq 5′-GACTACHVGGGTATCTAATCC-3′ [47]. On the other hand, PCR amplification of the whole region of the 16S rRNA gene (~1500 bp) was performed on the egg samples using fusion primers 27F 5′-AGRGTTTGATCMTGGCTCAG-3′ and 1492R 5′-GGTTACCTTGTTACGACTT-3′. The PCR reactions based on both sets of primers were performed in 25 μL reactions containing KAPA Taq Buffer (10×) at a final concentration of 1×, dNTP mix solution at 200 μM each, forward and reverse primer solution at 0.4 μM, 0.5 U of KAPA Taq DNA polymerase (5 U/μL), ≤250 ng from the template DNA solution, and sterile deionized water. The amplification process involved initial denaturation for 5 min at 95 °C and 35 cycles of denaturation for 30 s at 95 °C, annealing for 30 s at 54 °C, and extension for 30 s/500 bp at 72 °C, followed by 5 min of final extension step at 72 °C. Both positive and negative controls were present in parallel. To examine the presence and size of the amplified fragments, PCR products were electrophoresed on 1.5% (*w*/*v*) agarose gels in TAE buffer (1×) (40 mM Tris-acetate, 1 mM EDTA). Bio-Rad’s Gel DocTM XR+ was used to visualize the amplified products of around 550 bp and 1450 bp for V3–V4 and 16S regions, respectively. Positive PCR products of the correct size were then purified using polyethylene glycol (20% PEG, 2.5 M NaCl) [48].

### 2.3. Libraries Preparation and Sequencing

#### 2.3.1. Illumina MiSeq Sequencing of 16S rRNA Amplicons (V3–V4 Region)

To incorporate the indexes and the Illumina adaptors in the larvae, rearing water, and feed samples, a second PCR was carried out in a final volume of 50 μL. Each reaction contained 10 μL of KAPA Taq Buffer (10×), 0.4 μL of dNTPs solution (25 mM each), 5 μL of the forward/reverse indexing primer (10 μM), 0.2 μL of KAPA Taq DNA Polymerase (5 U/μL), and 2 μL of the diluted purified PCR product (10 ng/μL) and sterile deionized water. The temperature profile used for the amplification was as follows: 3 min of initial denaturation at 95 °C and 8 cycles of denaturation at 95 °C for 30 s, 30 s of annealing at 55 °C, and 30 s of extension at 72 °C, and finally, 5 min of final extension at 72 °C. The resulting amplicons were purified using the NucleoMag NGS Clean-up and Size Selection kit (Macherey-Nagel, Düren, Germany) based on the manufacturer’s instructions. Using a Quawell Q5000 micro-volume UV-Vis spectrophotometer, indexed amplicons from different samples were quantified and pooled in equimolar ratio (8 nM). High-throughput amplicon sequencing was performed using a 2 × 300 bp paired-end kit on an Illumina MiSeq platform by Macrogen (Seoul, Republic of Korea).

#### 2.3.2. ONT MinION Sequencing of 16S rRNA Amplicons (V1–V9 Region)

Library preparation for the amplified full 16S region in egg samples was performed using the Native Barcoding Kit 96 V14 (SQK-NBD114.96, ONT, Oxford, UK) following the manufacturer’s recommendation. Briefly, 200 ng of the purified full-length 16S purified PCR product of each sample was used as starting material for the library preparation. The final library with approximately 35 fmol of barcoded DNA was loaded into a MinION R10.4.1 flowcell (FLO-MIN114, ONT, Oxford, UK). Sequencing was carried out for 48 h on a MinION Mk1B device (ONT, Oxford, UK). The sequencing device, data acquisition, and real-time basecalling were monitored via the MinKNOW software (version 23.11.5, Dorado v7.2.13) with the high accuracy option using default settings.

### 2.4. Bioinformatic Data Processing

#### 2.4.1. Illumina Sequencing Data

Raw sequencing reads were demultiplexed, trimmed of Illumina adapters, and converted to FASTQ using the Illumina standard algorithm. A combination of USEARCH version 11 [49] and QIIME2 distribution 2019.1 [50] was used for the analysis and processing of the resultant sequences. Using the fastq_mergepairs command, the forward and reverse reads from each sample were assembled into paired-end fragments and merged into a single Fastq file. Fragments with less than 400 bp length were excluded from the analysis. The fastq_filter command was used to enhance the constructed sequence quality, while the fastx_uniques tool was used to eliminate duplicate sequences. Unique paired-end fragments were then clustered into operational taxonomic units (OTUs) with cluster_otus command based on 97% OTU clustering using the UPARSE algorithm [51]. Using the uncross command, cross-talk errors were identified and eliminated using the UNCROSS2 algorithm [52]. Using the otutab_trim command, rare OTUs (less than 0.1% of all sequences in all samples) were discarded. Using the BLAST+ algorithm [53] as implemented in QIIME2, taxonomy was assigned to the representative sequences of the discovered OTUs based on the 16S rRNA gene SILVA 138.2 release database [54]. The phylogenetic tree was constructed using the FastTree algorithm [55] and rooted using midpoint-root approach as implemented in QIIME2.

#### 2.4.2. Nanopore Sequencing Data

The adapter and barcode sequences were trimmed using Porechop v0.2.4 [56]. The reads were filtered by size and quality using NanoFilt (v.2.8.0-1) [57], retaining 1200–1800 bp sequences with a mean quality score > 8. Filtered reads from different samples were then concatenated into a single Fastq file. Subsequent analyses were performed using a modified NanoClust pipeline [58]. Briefly, unsupervised read clustering was performed with UMAP v0.5.7 [59], and HDBSCAN v0.8.40 [60]. A representative read was then selected from each cluster and polished using racon v1.4.3 [61] and medaka v1.12.0 [62] based on the remaining reads of the same cluster. Taxonomic classification of polished sequences was performed against the SILVA database, release 138 [63], using the classify-consensus-blast feature as implemented in the QIIME2 pipeline [50].

### 2.5. Bacterial Composition and Diversity Analysis

Alpha diversity indices, as well as indices depicting the population structure, were calculated using the vegan R package [64] based on a normalized OTU table at a depth of 5000 sequences/sample (ACE, Chao1, Shannon, and Simpson). The OTU count table was used to calculate the good’s coverage. Pairwise ANOVA was used to identify significant differences in alpha diversity indices between the different groups.

The diversity across samples (beta-diversity) was estimated based on Generalized UniFrac distance using the GUniFrac R package [65]. Overall community structure differences and similarities were presented using constrained ordination approaches (Canonical analysis of principal coordinates, CAP) based on 999 permutations, and unconstrained ordination approaches (Principal coordinates analyses, PCoA). Metaxplore (http://metaxplore.eu/, accessed on 1 February 2025) was used to conduct and illustrate CAP, PCoA analyses, and PERMANOVA tests [66]. Statistical significance was described by a *p*-value of equal to or less than 0.05.

## 3. Results

### 3.1. Dataset Overview

Sequencing of the hypervariable regions V3–V4 of the 16S rRNA gene resulted in 7,409,362 raw reads. Following quality filtering and eliminating chimeric sequences, 5,538,269 high-quality reads were grouped into OTUs, with an average of 19,850 reads/sample. With a mean coverage estimate of 0.98, the microbial communities were well sampled. After removing OTUs with an abundance of less than 0.1% across all samples, 90 OTUs were classified into seven phyla, nine classes, and 71 genera (Table S1). Pseudomonadota was the most abundant taxonomic group in all the studied samples (average 73.2 ± 1.5%), followed by Bacteroidota and Cyanobacteriota, representing around 12.0 ± 1.0% and 10.1 ± 0.8% of the total bacterial community. At the class level, Pseudomonadota was represented by Gammaproteobacteria and Alphaproteobacteria (45.1 ± 2.4% and 28.12 ± 1.55%, respectively), Bacteroidota with Bacteroidia (12.1 ± 1.0%), and Cyanobacteriota with Cyanobacteriia (10.1 ± 0.85%). Various taxa were detected at the genus level, with *Psychrobacter*, *Chlorella* (the chloroplast of the eukaryotic microalgae), and *Pseudophaeobacter* being the most dominant, followed by *NS3a-marine-group*. Each of the remaining genera showed less than 4% average relative abundance across all samples.

Regarding the eggs samples, the sequencing of the full 16S rRNA gene resulted in a total of 876,146 raw reads. After quality filtering, 269,864 high-quality reads were obtained with an average of 7496 reads/sample. After removing the clusters with an abundance of less than 0.1% across all samples, 137 clusters were categorized into eight phyla, 10 classes, and 74 genera (Table S1). Pseudomonadota was the most abundant taxonomic group in egg samples (66.50 ± 4.10%), followed by Bacillota, which represented 22.9 ± 4.6% of the total bacterial community. At the class level, Pseudomonadota was represented by Gammaproteobacteria and Alphaproteobacteria (61.7 ± 2.8% and 4.8 ± 0.7%, respectively) and Bacillota with Clostridia and Bacilli (16.9 ± 2.9% and 6.0 ± 0.8%). Various taxa were detected at the genus level, with *Psychrobacter* (33.6 ± 3.4%) being the most dominant, followed by *Vibrio* (15.6 ± 2.7%). Each of the remaining genera showed less than 10% relative abundance.

### 3.2. The Diversity of the Bacterial Communities Based on the Disinfection Method

**Figure 1:**
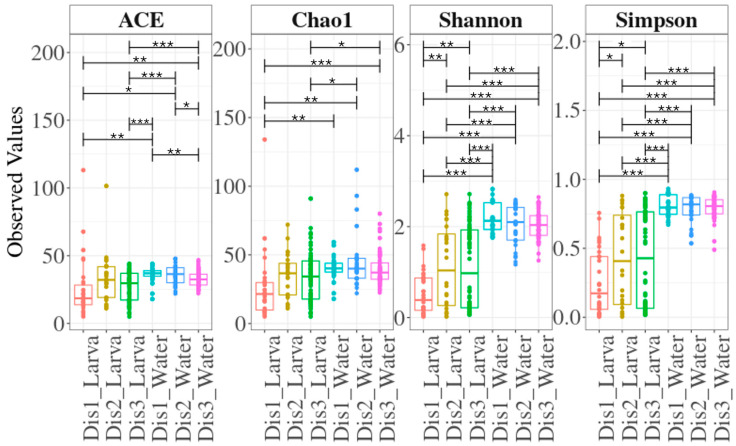
Species richness and diversity indices for larval and water samples based on the disinfection method. Boxes represent the interquartile range, the line within the boxes is the median, and the dots represent samples. Lines with asterisks above the boxes denote significant differences between compared pairs of samples. Asterisks denote different *p*-value ranges (***: *p* ≤ 0.001; **: 0.001 < *p* ≤ 0.01; *: 0.01 < *p* ≤ 0.05).

Overall, larval samples exhibited lower species richness and diversity compared to their rearing water (Figure 1). Larval samples within the three types of disinfection showed, to some extent, similar bacterial richness. However, samples reared in tanks disinfected with the Dis1 method exhibited significantly lower bacterial diversity compared to those reared in tanks disinfected with the Dis2 and Dis3 methods. On the other hand, overall, no significant differences were observed in the richness and the diversity within the rearing water samples, except for the ACE index, which showed a difference between Dis1 and Dis3 (Figure 1).

**Figure 2:**
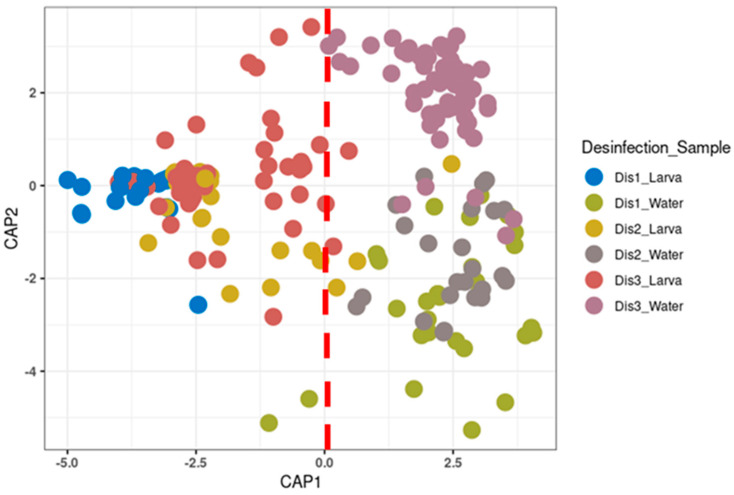
Canonical analysis of principal coordinates (CAP) of beta-diversity based on GUnifrac distance between each type of disinfection at different components of the larviculture (*p*-value: 0.001).

As evident from the CAP and PCoA, along with PERMANOVA analysis, the bacterial profile of the larvae reared in tanks disinfected with the Dis1 method showed a significant difference compared to those reared in tanks disinfected with the Dis2 and Dis3 methods (PERMANOVA, *p* < 0.05; Figure 2 and Figure S1). However, the change in the dry period from 1 week (Dis2) to 50 days (Dis3) did not affect the bacterial community of larvae (PERMANOVA, *p* = 0.465). On the other hand, the disinfection method affected, to some extent, the bacterial profile of the rearing water (PERMANOVA, *p* < 0.05; Figure 2).

### 3.3. Taxonomic Composition of Bacterial Communities Based on the Type of Disinfection

**Figure 3:**
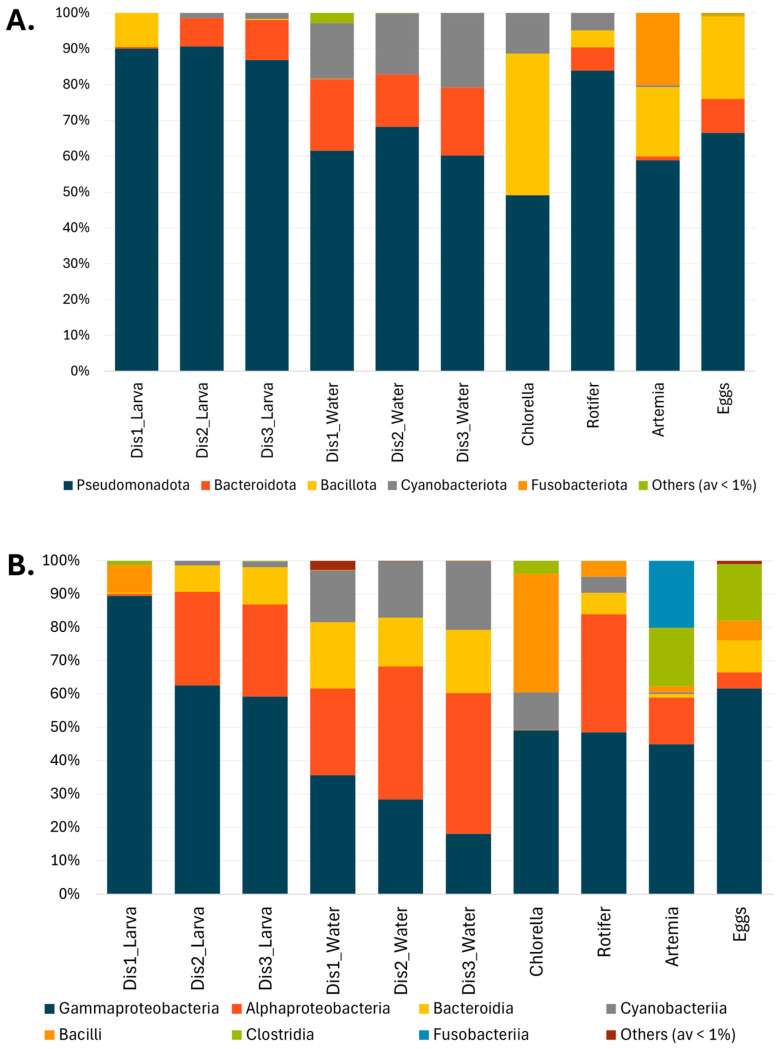
The microbial composition associated with the three different types of disinfection, Dis1, Dis2, and Dis3, in *S. aurata* larvae, rearing water, feed, and eggs (**A**) at the phylum level, and (**B**) class level.

**Figure 4:**
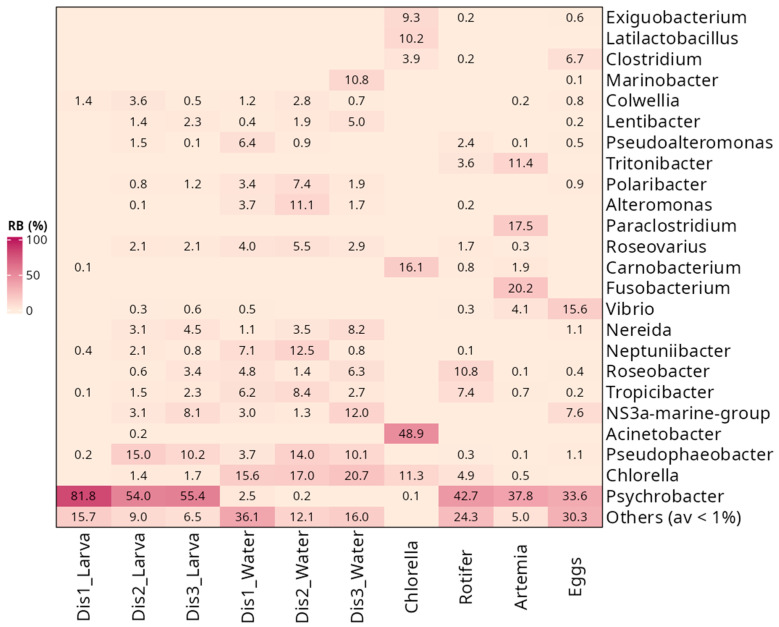
Comparison of microbial composition associated with three types of disinfection, Dis1, Dis2, and Dis3, in *S. aurata* larvae, rearing water, and feed from all larval feeding stages at the genus level.

The bacterial community of larval samples was characterized mainly by the presence of four phyla: Pseudomonadota, Bacillota, Bacteroidota, and Cyanobacteriota (Figure 3A). Pseudomonadota was the dominant phylum in larvae, representing 90% of the bacterial community. In larvae from Dis1 tanks, the most abundant OTUs belonged to Gammaproteobacteria (89.4 ± 2.3%), followed by Bacilli (8.2 ± 2.2%) (Figure 3B). Larvae reared in Dis2 and Dis3 tanks shared similar bacterial communities, characterized by a decrease in Gammaproteobacteria (62.6 ± 4.5%, and 59.2% ± 5.0%, respectively) and an increase in Alphaproteobacteria (28.1 ± 3.7%, and 27.6 ± 3.5%, respectively) and Bacteroidia (7.9 ± 1.6%, and 11.2 ± 2.1%, respectively) (Figure 3B). At the genus level, larvae reared in Dis1 tanks showed a dominance of *Psychrobacter* (81.8 ± 2.8%) (Figure 4). For Dis2 and Dis3 tanks, larvae showed a lower abundance of *Psychrobacter* compared to those in Dis1 tanks (54.0 ± 5.3% and 55.4 ± 5.3%, respectively), followed by *Pseudophaeobacter* (15.0 ± 2.3% and 10.2 ± 1.7%, respectively). The relative abundance of each of the remaining genera was less than 10% (Figure 4, Table S1).

At a higher taxonomic level, similarities were observed in the bacterial community of the rearing water. At the phylum level, Pseudomonadota were dominant regardless of the disinfection method used (61.6 ± 3.2%, 68.3 ± 2.3%, and 60.2 ± 2.5% for Dis1, Dis2, and Dis3, respectively) (Figure 3A). At the class level, the predominance of Gammaproteobacteria (35.7 ± 3.9%) over Alphaproteobacteria (25.9 ± 1.4%) was observed in rearing water from tanks disinfected with Dis1, followed by Bacteroidia and Cyanobacteriia (19.9 ± 2.9% and 15.6 ± 2.1%, respectively) (Figure 3B). Water from the Dis2 and Dis3 tanks showed a decrease in Gammaproteobacteria (28.4 ± 3.9% and 18.1 ± 3.8%, respectively) along with an increase in Alphaproteobacteria (39.9 ± 2.6% and 42.2 ± 2.1%, respectively). They also contained Bacteroidia (14.6 ± 2.6% and 19.0 ± 1.6%, respectively) and Cyanobacteriia (17.0 ± 1.6%, and 20.7 ± 1.8%, respectively) at comparable values to Dis1 (Figure 3B). At the genus level, diverse communities dominated by *Chlorella* were found in rearing water regardless of the disinfection technique (15.6 ± 2.1%, 17.0 ± 1.6%, and 20.7 ± 1.8% in Dis1, Dis2, and Dis3, respectively). Water from Dis2 tanks was also characterized by increased density of *Pseudophaeobacter* and *Neptuniibacter* (14.0 ± 1.4% and 12.5 ± 1.5%, respectively). On the other hand, the rearing water from Dis3 tanks showed increased numbers of NS3a marine group and *Pseudophaeobacter* (12.0 ± 1.6% and 10.1 ± 1.3%, respectively) (Figure 4).

The bacterial taxa present in the larval feeds were found to be diverse. Samples from all the larval feeding stages were included in the survey, including *Chlorella*-based feed, rotifers, and *Artemia nauplii*. *Chlorella*-based feed was dominated by members of Pseudomonadota, mainly by *Acinetobacter* (48.9 ± 4.4%), followed by Bacillota, namely *Carnobacterium* (16.1 ± 2.9%), and *Latilactobacillus* (10.2 ± 3.1%) (Figure 4, Table S1). Rotifers were also dominated by Pseudomonadota (80.3%), specifically by *Psychrobacter* (42.7 ± 5.7%), in addition to a minor community of *Roseobacter* (10.8 ± 2.5%) and *Tropicibacter* (7.4 ± 1.0%). Similarly, *Artemia nauplii* were characterized by the strong presence of Pseudomonadota, mainly by *Psychrobacter* (37.8 ± 4.6%), followed by *Fusobacterium* (20.2 ± 4.2%), a member of the phylum Fusobacteriota, and *Paraclostridium* (17.5 ± 3.7%).

Samples from eggs were also collected to examine whether any genera persisted or were lost from the egg to the larval stage. The eggs were also mainly dominated by members of the class Gammaproteobacteria, with more than 60% relative abundance (Figure 3B). More specifically, the most frequently observed genera were *Psychrobacter* (33.6 ± 3.4%) and *Vibrio* (15.6 ± 2.7%). Notably, *Psychrobacter* was still dominant even in the larval stage, while *Vibrio* had decreased dramatically (Figure 4).

## 4. Discussion

Farmed fish are in constant interaction with their microbiome, which plays a vital role in determining their overall health and well-being [14,67,68,69]. In many studies, it has been stated that the microbiome of farmed fish is shaped by multiple factors, including water quality and feed composition [15,70,71,72,73,74,75,76,77]. Another important factor is the disinfection protocols applied to the tanks into which the hatched eggs will be introduced to start the larval life cycle. In recent years, various studies have been carried out on the disinfection methods used in aquaculture. Usually, the effect of chemical disinfectant agents is examined either on fish larvae, on the rearing water, or even on their feed [78]. Multiple studies have concluded that the concentration of disinfectants and the duration of their application are crucial for their effectiveness in eliminating pathogens in aquaculture facilities [78,79].

Here, we examined the bacterial communities associated with seabream larvae and their associated rearing water after the application of three different disinfection techniques in the rearing tanks. We additionally assessed the bacterial communities of larval feed and eggs before they were placed in the disinfected tanks. The current analysis, which makes use of next-generation sequencing of 16S rRNA amplicons, shows that disinfectants have a significant impact on the bacterial communities of *S. aurata* larvae. Distinctive features are evident between the larval microbiome from Dis1 versus Dis2, and Dis3, which have common characteristics. Furthermore, despite the differences observed between the bacterial community of larvae and the remaining vital components of the larviculture ecosystem, including the rearing water, feed, and eggs, similar traits were also recorded.

### 4.1. Type of Disinfection and Microbial Communities

The variation observed between the bacterial communities of the three studied larval groups suggests that the microbial composition was influenced by the type of disinfection that was applied to the tanks. Since the larval-associated microbiome is shaped by a variety of other factors, such as the rearing water and the provided live feed [12,80,81,82,83], we tried to ensure uniformity in all the tanks used in this study by standardizing the aforementioned factors. Despite the regular use of disinfectants in aquaculture [78,84], most studies focus mainly on adult fish, examining the effect of different dosages and their prolonged application in rearing water [35,79]. To the best of our knowledge, no studies have specifically investigated the impact of tank disinfection on the microbial community of fish larvae. This gap in research is especially relevant given the widespread use of disinfectants in this stage of aquaculture production.

Three main phyla were identified by the taxonomic classification of the microbiome of *S. aurata* larvae: Pseudomonadota, Bacteroidota, and Bacillota, with variations in the relative abundance across disinfection methods. In previous studies, Pseudomonadota also appeared to be the dominant phylum of the bacterial community found in both European seabass and seabream eggs and larvae [85,86]. The bacterial communities of the larvae in all types of disinfection were mainly dominated by members of Gammaproteobacteria, and more specifically, the genus *Psychrobacter*. This genus appears to be mostly part of the normal surface microbiota of fish skin, seaweeds, or algae, but it can also be found in the tissues of marine animals and sponges [87,88,89].

The commercial PAA-HP (Dis1) and PMS-SC (Dis2 and Dis3) mixtures are commonly used in aquaculture for water treatment, facility disinfection, and control of bacterial or parasitic infections during disease outbreaks [78,90,91,92]. They differ in the way they impact the water chemistry, which may affect the larviculture environment and therefore the overall health of larvae. PMS used in Dis2 and Dis3 produces highly reactive sulphate radicals (SO_4_^−^) and hydroxyl radicals (^•^OH), which target bacteria, fungal spores, and viruses [93,94,95]. As a gram-negative bacterium, *Psychrobacter* may be susceptible to these radicals, which can damage proteins, the cell wall, and the cell membrane [93]. This effect may explain the lower abundance of *Psychrobacter* in larval samples from rearing tanks disinfected with Dis2 and Dis3. The reduction in the presence of *Psychrobacter* may have led to the proliferation of other bacterial genera, thus increasing the recorded diversity in these tanks. Similarly, PAA-HP used in Dis1 also have strong antimicrobial properties. However, their action is often milder than that of potassium peroxymonosulfate, which was used in Dis2 and Dis3 tanks [96,97,98]. In this regard, the cultivable bacterial diversity of the shrimps abdominal cavity has been shown to remain unaffected by different doses of PAA in the rearing water [79]. The milder effect of PAA-HP potentially allows *Psychrobacter* to survive in higher abundance in larvae reared in tanks disinfected with Dis1 and dominate the community. However, since these compounds rarely exhibit specialized activity, it is more likely that the application of Dis1 resulted in the drastic reduction in bacterial diversity in the larvae, leaving behind a rather simple community composed mainly of *Psychrobacter* and the tolerant gram-positive genus *Bacillus*. Significant reduction in the bacterial diversity of the external surface of shrimps was previously recorded 29 days after the application of PAA (1 mg/L) in the rearing water [79]. In this scenario, the other two disinfection methods had a milder effect on the community of their larvae and eventually led to the formation of more diverse and possibly more resilient bacteriomes. These bacteriomes were characterized by the presence of multiple bacterial genera, including *Pseudophaeobacter*, *Trobicibacter*, NS3a marine group, *Roseobacter*, etc., that were generally absent or found with very low abundances in Dis1. This notion is also supported by the diversity of the microbiota of the water that resembled more the microbiota of Dis2 and Dis3 larvae. Regardless of the scenario, it seems that *Psychrobacter* is a rather persistent genus capable of retaining a strong presence in larvae, possibly assisted by its availability in the microbial pools of the eggs and the feed, mainly rotifer and *Artemia*.

### 4.2. Factors Influencing the Composition of the Larval Microbiota

The microbial community observed in rearing water was different from larvae, with higher richness and diversity in all the disinfection types. It was dominated by members of Cyanobacteriia and Alphaproteobacteria, such as the genera *Chlorella* (the chloroplast of the eukaryotic microalgae) and *Tropicibacter*, respectively. Despite the observed differences in the dominant genera, water samples and seabream larvae, especially Dis2 and Dis3 larvae, shared common characteristics, including the presence of *Tropicibacter*, NS3a marine group, *Roseobacter*, *Neptuniibacter*, *Nereida*, *Polaribacter*, *Lentibacter*, and *Colwellia*. The bacterial communities of the water seem to be affected by all the components of the tank ecosystem. They may receive bacteria from the disinfected tank walls, the eggs that are placed inside the tanks to hatch, the feed that is provided to the larvae, and ultimately from the developing larvae. For example, water samples seem to have received *Chlorella* from the larval feed (mostly *Chlorella* and rotifers), as well as *Tropicibacter* and *Roseobacter* from the rotifers. At the same time, water also affects the microbiome of larvae. During development, the larvae tend to acquire bacteria from the rearing water, such as *Pseudophaeobacter*, which is consistent with previous studies on *S. aurata* larvae [42,43]. Notably, water samples contained a very small amount of *Psychrobacter*, even though the bacterium was prevalent in feed, eggs, and larvae. This discrepancy is probably due to the fact that the identified *Psychrobacter* strains are either symbiotic or strongly associated with their hosts, even though free-living species are known to exist (e.g., in seawater) [99]. Another putative reason for the low density of *Psychrobacter* is its interaction with *Chlorella* in the water, as the eukaryotic microalgae are highly competitive with certain bacteria, such as *Bacillus* and *Pseudomonas* [100]. This interspecific competition could be further supported by the fact that the bacterium was not identified in the *Chlorella*-based feed. On the contrary, the possibility that this depletion could be attributed to disinfectant residues in the water that affected the proliferation of *Psychrobacter* seems rather low. Similarly, it is difficult to attribute the remaining observed differences between the microbiota of the water samples to the application of the three disinfection methods on the tanks. As mentioned previously, these communities are rather rich and diverse, and the observed variations in relative abundance could be most likely linked to the dynamic interactions among their components.

A detailed examination of the developmental stages from egg to larva allows for the estimation of which bacterial taxa are eliminated, and which persist throughout early growth. The bacterial profile of the eggs revealed the presence of the genera *Clostridium*, *Pseudomonas*, and *Vibrio*, known to include species pathogenic to fish [101,102]. In contrast, these genera were either absent or nearly undetectable in larvae. The discrepancy between the two developmental stages can also be influenced by the sequencing approach: the bacterial community in eggs was characterized using full-length 16S rRNA sequencing, whereas the larvae were analyzed only using the V3–V4 region. Since less abundant taxa can be masked when using Illumina V3–V4 sequencing, this limitation may have contributed to the observed differences [103]. On the other hand, *Psychrobacter* seems to persist from the eggs to the larval stage, where it emerges as the dominant genus in both developmental stages. This aligns with the findings identifying *Psychrobacter* as a common colonizer of the gastrointestinal tracts of finfish species [104]. Overall, it seems that the bacterial profile of the eggs influenced the bacterial communities of both larvae and water samples.

In larviculture systems, *Chlorella*, rotifers, and *Artemia nauplii* are frequently employed as feed organisms. They sustain the early growth and development of fish larvae and offer vital nutritional elements [105]. Both the current and previous studies have shown that there is a significant overlap between the larval microbiome and live feed [42,43,83]. Regarding *Chlorella*, its effect on the bacterial profile of the larvae was negligible since the observed dominant genera, such as *Acinetobacter* and *Carnobacterium*, were not present in the larvae samples. On the contrary, the bacterial profiles of rotifers and *Artemia nauplii* harbored common taxa with the larvae, as both categories were dominated by the genus *Psychrobacter* and contained *Pseudophaeobacter*, *Tropicibacter*, *Roseobacter*, *Vibrio*, and *Pseudoalteromonas*. The presence of *Vibrio* in rotifer and *Artemia*-based feed may indicate a source of potentially pathogenic strains, as several species have been implicated in causing disease in farmed fish [106]. In this regard, *Vibrio* was identified with very low density in Dis2 and Dis3 larvae. However, its presence in the larvae may also be linked to the bacterial pool of the eggs, which contained a high titer of *Vibrio*. Additionally, certain genera, such as *Ruegeria*, *Paraclostridium*, and *Fusobacterium*, were identified exclusively in these feed categories. It seems that these genera do not proliferate in the water or larvae, possibly due to competitive interactions with the remaining bacteria of the larviculture ecosystem, although an effect of disinfectant residues in the water cannot be excluded.

This study provides practical insights relevant to commercial hatcheries using similar disinfection protocols for seabream. However, due to its limited taxonomic, geographical, and methodological breadth, generalizability is constrained to other species, other systems, or different microbial endpoints (functional or virulence-related microbial properties may not follow the same trends as taxonomic composition). While the study has methodological and contextual limitations (no functional microbiome analysis, lack of host health, single-species and single site experiment), it makes a meaningful contribution to aquaculture microbiome research. It underscores the complex interplay between disinfection practices, microbial ecology, and hatchery outcomes. Future work building on this foundation—particularly with functional analyses—could lead to microbiome-informed hatchery management strategies that enhance larval health and performance while minimizing unnecessary interventions.

## 5. Conclusions

This study demonstrated that that *S. aurata* larvae reared in tanks treated with Dis1 were characterized by distinct bacterial communities compared to their counterparts reared in tanks treated with Dis2 and Dis3. Members of the class Gammaproteobacteria dominated the bacterial profiles of larvae, with most associations linked to the genus *Psychrobacter*, which was also abundant in egg samples. Water samples developed different bacterial communities devoid of *Psychrobacter*, despite the strong presence of the bacterium in most components of the larviculture ecosystem. Nonetheless, they shared multiple bacterial genera among them, but also with larvae from tanks subjected to Dis2 and Dis3. Moreover, *S. aurata* larvae exhibited an increased likelihood of obtaining bacteria from the supplied live feed, mainly rotifers and *Artemia nauplii*, including *Psychrobacter*, *Tropicibacter*, and *Roseobacter*. However, many prevalent genera that were identified in the feed did not establish in either the water or the larvae. Overall, these findings suggest that the microbial profiles of the larvae were influenced by both their interactions with the other components of the tank ecosystem, and by the specific disinfection methods applied. However, the functional contribution of key microbial taxa, such as *Psychrobacter*, to the host remains unclear. Future studies are required to evaluate the roles of these species in *S. aurata* performance and to assess the long-term effects of the different disinfection methods on the microbiota at later life stages of *S. aurata*.

## Figures and Tables

**Table 1:** Larval and water samples used in the 16S rRNA amplicon sequencing survey.

Disinfection	Active Ingredient	Application	Dry Period	No. of Tanks	No. of Samples	
					Larva	Water
Dis1	PAA-HP	Nebulization	0 days	2	30	30
Dis2	PMS-SC	Wash	7 days	2	30	30
Dis3	PMS-SC	Wash	50 days	4	60	60
Total				8	120	120

## Data Availability

The NCBI Bioproject accession number for the raw sequencing data reported in this study is PRJNA1113936.

## Supplementary Materials

The following supporting information can be downloaded at https://www.mdpi.com/article/10.3390/microorganisms13061359/s1, Figure S1: Principal Coordinate Analysis (PCoA) of beta-diversity based on GUnifrac distance between each type of disinfection at different components of the larviculture.; Table S1: Identified taxa in *S. aurata* larvae, rearing water, feeds, and eggs.
