# Supplementary figures and images for: The Impact of Tank Disinfectants on the Development of Microbiota in Gilthead Seabream (Sparus aurata) Larviculture Systems
Source: Microorganisms. 2025 Jun 11;13(6):1359. doi: 10.3390/microorganisms13061359 (PMC12195472; doi:10.3390/microorganisms13061359)

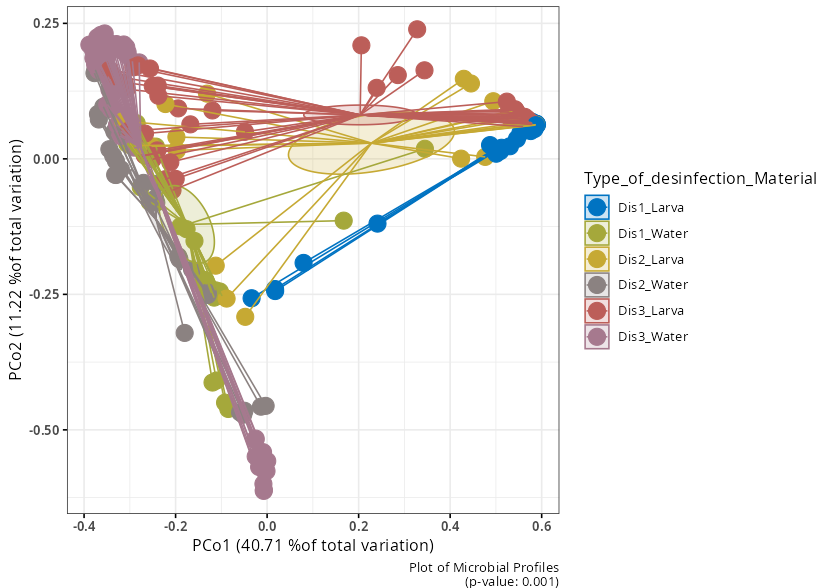

Supplement: Supplementary file 1 [file microorganisms-13-01359-s001.zip › microorganisms-3660432-supplementary/SUPPLEMENTARY/FigureS1_PCoA.png]
